# Influenza evolution navigates stability valleys

**DOI:** 10.7554/eLife.00842

**Published:** 2013-05-14

**Authors:** Mary M Rorick, Mercedes Pascual

**Affiliations:** 1 **Mary M Rorick** is at the Department of Ecology and Evolutionary Biology, University of Michigan, Ann Arbor, United Statesrorick@umich.edu; 2 **Mercedes Pascual** is at the Department of Ecology and Evolutionary Biology, University of Michigan, Ann Arbor, United Statespascual@umich.edu

**Keywords:** epistasis, protein evolution, protein instability, influenza, Viruses

## Abstract

By reconstructing how an influenza protein collected in 1968 might have evolved into one collected in 2007, researchers have obtained new insights into the interactions between genetic mutations.

**Related research article** Gong LI, Suchard MA, Bloom JD. 2013. Stability-mediated epistasis constrains the evolution of an influenza protein. *eLife* **2**:e00631. doi: 10.7554/eLife.00631

**Image** Reconstructing the evolutionary path of an influenza protein

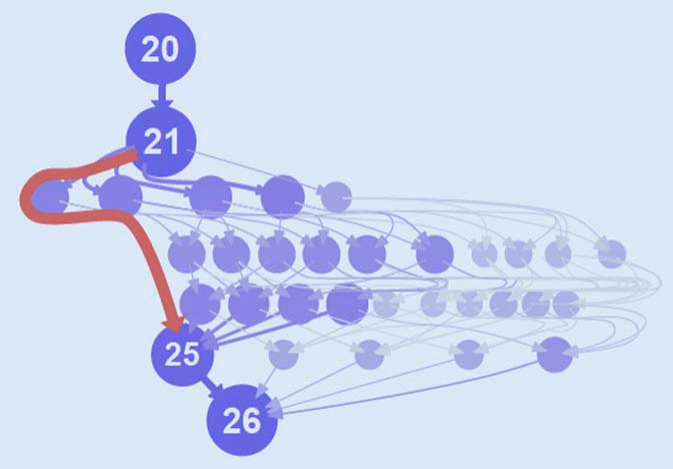

An individual’s phenotype is shaped by a multitude of interactions: those among genes and those with the environment. This means that a mutation’s fitness, and likelihood of evolutionary success, is largely a function of the specific genetic background and environment in which it resides. For example, a mutation that is advantageous within one genome may be detrimental or even lethal in another. This occurs because the effects of a gene on an organism’s phenotype often depend on the actions of many other genes—a phenomenon known as epistasis. Complex epistatic interactions limit the pathways along which evolution can proceed by natural selection. Now, in *eLife*, Lizhi Gong, Marc Suchard and Jesse Bloom—at the Fred Hutchinson Cancer Research Center and the University of California Los Angeles—provide new insights into a remarkably simple mechanism that can explain many of the interactions that appear to be shaping the evolutionary path of a human influenza virus protein (Gong et al., 2013).

Several decades ago, John Maynard Smith and others predicted that mutation fitness effects should be highly contingent on the genomic context in which they occur, and they described these predictions using the concept of a fitness landscape. They observed that when the selective effect of a point mutation is highly dependent on a protein’s genotype, the fitness landscape is not smooth with a single peak; rather, it is rough and contains various local peaks, with valleys in between. Because natural selection avoids these valleys and instead climbs local peaks in the fitness landscape, a rough terrain implies path dependency: not all evolutionary trajectories lead to the same place. Out of this work came the important prediction that evolution proceeds in a way that is strongly constrained by the order in which mutations occur (Maynard Smith, 1970; Kimura, 1985).

Recently, it has become possible to study these processes experimentally. Data sets of genetic sequences for fast-evolving viruses sampled for many time points make it possible to recapitulate with confidence the chain of mutations that separates any two protein variants. Moreover, advances in site-directed mutagenesis allow historical protein variants to be recreated, so that their fitness can be compared with that of existing proteins.

Gong et al. use computational modelling to infer the most likely evolutionary paths between two variants of the influenza nucleoprotein: one collected in 1968 and the other in 2007. In most cases, they are also able to determine the order in which the mutations occurred. Next, they introduce each of these mutations, one at a time, into the original protein sequence. They also recreate the intermediate proteins that arose at each stage of the evolutionary path. By comparing the fitness of each mutated protein with that of the corresponding natural intermediate in which the mutation first appeared, they manage to identify three mutations that would have been deleterious for the original protein, but have no adverse impacts on the natural intermediates: such mutations are said to be epistatically constrained.

Prior studies have anecdotally demonstrated the existence of such mutations in natural protein evolutionary paths. However, Gong et al. go further by investigating nearly all naturally occurring mutations along a relatively long evolutionary path (consisting of 39 steps), and by examining the causes of these epistatic interactions. They determine the stability of each nucleoprotein variant by measuring its melting temperature, with higher melting temperatures corresponding to more stable proteins (Figure 1). They find that—with one exception—the combined effect of a group of mutations on the fitness of a protein can be explained in terms of the individual effect of each mutation on the stability of the protein.

**Figure 1.**
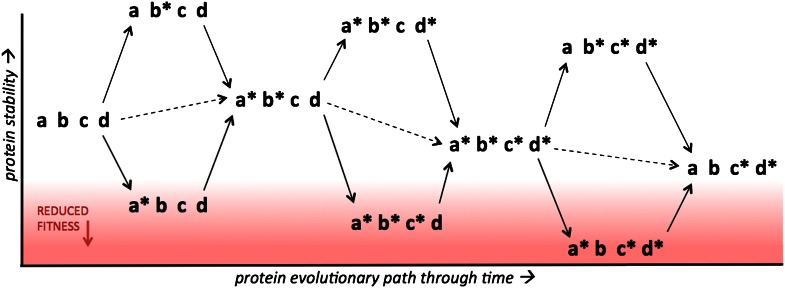
Protein stability determines the accessibility of multiple possible evolutionary paths separating two protein variants. The results of Gong et al. suggest that increasing the stability of a protein (y axis) above a certain threshold has no effect on fitness, which means that protein evolution can occur along multiple alternative routes. However, fitness decreases rapidly when stability drops below the threshold (red region), in which case evolution will select for mutations that increase stability. Four sites within a protein are shown, with two possible states for each site, indicated by the presence/absence of the asterisk. Single point mutations are indicated by solid arrows, double mutations are indicated by dashed arrows. Consider the case where a* and c* are mutations that help a protein to evade the immune system and, like the great majority of random mutations, they reduce the stability of the protein relative to the parent protein (abcd). However, mutations that increase stability, such as b* and d*, can compensate for mutations that reduce stability, which means that several proteins containing a* and/or c* can remain above the stability threshold required for optimal fitness.

Gong et al. show that all three of the epistatically constrained mutations reduce the stability of the protein, and that all were preceded by, or occurred almost concurrently with, one or more mutations that increase stability. This lends support to the classic models for how evolution should occur when the fitness landscape contains various valleys and peaks (Figure 1; Maynard Smith, 1970; Kimura, 1985). In other words, mutations that increase the stability of the influenza nucleoprotein enable it to tolerate other mutations that decrease its stability, and this phenomenon appears to account for most of the epistasis they observe.

The results also suggest that once the protein reaches a threshold level of stability, mutations that increase the stability further have no effect on fitness. This is in contrast to the results of a related study (DePristo et al., 2005), and it suggests that extra stability might accumulate through neutral evolutionary processes. Epistatically constrained mutations also seem to have a disproportionately large role in helping proteins escape detection by the immune system. This is suggested by the fact that the three epistatically constrained mutations in influenza nucleoprotein occur more frequently in immune system targets than do the other mutations in the evolutionary path.

The results of Gong et al. paint the following picture of nucleoprotein evolution: natural selection maintains stability above the minimum threshold required for a protein to be viable, but evolution allows for occasional mutations that increase the stability well above this threshold. While this extra stability has no immediate consequences for fitness, it does make a wide range of destabilizing mutations newly accessible to the protein, some of which could potentially contribute to immune escape. Selection will favour these variants if the stability of the protein remains above the threshold. However, if protein stability falls below the threshold, selection for stabilizing mutations will occur.

The work of Gong, Suchard and Bloom helps us move toward a better understanding of how epistasis shapes protein evolution. The insights gained from empirical studies such as these will aid the development of conceptual and computational models of evolution. This information will be particularly relevant for rapidly evolving viruses in the new field of ‘phylodynamics’, at the interface between phylogenetics and epidemiology (Grenfell et al., 2004). A tantalizing possibility is that mechanisms similar to the ones presented by Gong et al. underlie the immune escape of the protein hemagglutinin—a surface antigen for influenza subtype H3N2 and a major target of the human immune system (Koelle et al., 2006). Computational models in the field of phylodynamics have so far largely focused on mutations that influence immune escape but have little or no impact on other biological functions: there is a clear need to study the evolution of viruses while considering that each mutation may influence both the basic functioning of a protein as well as its appearance to the immune system. How evolution mediates such trade-offs is an open area of research that is informed by this study.

Gong et al. use a parent protein with the minimum stability naturally observed, and so their search for epistatically constrained mutations may be biased toward those that are destabilizing. Thus, it remains an open question whether or not other types of epistatic interactions are also important in shaping the evolutionary path of the influenza nucleoprotein, and of proteins in general.
